# Intercellular communication, NO and the biology of Chinese medicine

**DOI:** 10.1186/1478-811X-3-8

**Published:** 2005-05-18

**Authors:** Dina Ralt

**Affiliations:** 1 Izun & Tmura, Integrative Health Inst. 6 Nezach Israel st. Tel Aviv, Israel 64352

## Abstract

New multiple categories of health disciplines have become popular in the west and integration between the medicinal approaches has become essential.

The hypothesis presented here suggests a novel integrative view that combines Western biochemistry with the Chinese medicinal concept of qi.

The core for this hypothesis is that transmission of qi along the meridians is based on informational molecules that travel *via *an intercellular communication system. Acupuncture at specific points enhances the flow of the signaling molecules through this communication system.

Nitric oxide is suggested as a prime candidate for such a signaling molecule in the meridian system. The biochemistry of nitric oxide can shed light on the biology underlying Chinese medicine while Chinese medicinal data can provide a clue to the sought after framework for nitric oxide.

## Introduction

Recently there has been a great fervor in the health field around the inclusions of non-traditional disciplines [1]. For example, eastern medicines, naturopathy and healing have become popular and were recently introduced into medical school curricula. These changes have made Chinese medicine [2,3] attainable in the west just as Western medicine is to the east. However, open-mindedness [4,5] or legality [6,7] does not necessarily imply true integration between the medicinal approaches.

The aim of this article is to suggest a novel integrative view of the key concept in Chinese medicine, qi, meridians and acupuncture, with Western biochemistry. The crux of the hypothesis is that the transmission of qi along the meridians, involves informational molecules, which travel *via *an intercellular communication system, and, acupuncture at specific points enhances this communication system.

Nitric oxide (NO) is proposed here as a prime candidate for such a signaling molecule in the meridian system.

One analogy that can be made is to consider the word HELLO as an example to the signaling molecule. The information that HELLO transfers is not only the meaning of the word, which is HI, but mainly the way by which it is expressed e.g., loud, smiling, sad, laughing. If we think of NO in the context of a word like HELLO, it is suggested that the meaning of qi is not only a word like HI but rather an abundance of bodily information.

## Discussion

### Nitric Oxide

NO has been the most widely studied signaling molecule for more than a decade. It regulates blood pressure, contributes to the immune responses, controls neurotransmission and participates in cell differentiation and in many more physiological functions [8].

Nitric oxide (NO) the diffusible, signaling gas, is synthesized by 3 nitric oxide synthase isoforms (NOS): a neuronal (nNOS), inducible (iNOS) [9], or endothelial NO synthetase (eNOS) [10,11]. Using BH4 (tetrahydrobiopterin) [12] as a cofactor, these enzymes convert arginine to citruline and NO. The levels of NOS are altered by a variety of pathophysiological conditions such as hypertension, hypercholesterolemia, aging, cigarette smoking, diabetes, heart failure and under physical activity [13] and dementia [14].

NOS were also shown to be associated with a plethora of more than 20 proteins that affect the activity and spatial organization of NO synthesis within the cell. Some of these interactions may have functions beyond NO formation [15].

NO is such a crucial entity that the mammalian genome encodes three NOS genes with partially overlapping function that may compensate for each other if one of them is not functioning. For example, the essentiality and flexibility of the NO system allows nNOS enzyme to compensate for eNOS, in mutant mice lacking eNOS [16,17]. Recently NOS was shown to be essential for development (in Drosophila) by taking advantage of the fact that, unlike those of mammals, the genome of the fruit fly has only one NOS gene and is thus more amenable to mutagenesis [18].

### Qi, meridians and acupuncture

The idea of qi is fundamental to Chinese medical thinking. Qi is involved with all biological functions and circulates throughout meridians reaching the entire human body [19,20]. The goal of acupuncture as well as other Eastern medicinal methods (herbs, massage, Qigong) is to ensure the unobstructed flow of qi. When qi is strengthened or balanced, it can improve health and ward off or slow the progression of disease. Chinese medicine considers sickness or pain, a result of the qi blockage or unbalanced qi energy in the body [2]. Like NO, qi is essential for life, as Guanzi said [21] in 5^th ^century BCE, "When there is qi, there is life".

Much information has accumulated regarding the physiological effects of acupuncture. In 1998, a NIH panel concluded that acupuncture is an effective technique for relieving nausea and vomiting and an effective agent for relieving pain [22]. Needling of acupuncture points has subjective (needle sensation) and objective (e.g., serum cortisol increase or Ca(2+) oscillations) effects [23,24]. Acupuncture increases blood flow [25] and acupuncture points have high electrical conductance [26,27]. A relationship between acupuncture points and meridians to connective tissue planes [28] and perivascular space [29] has also been suggested. Possible mechanisms of acupuncture have been reviewed [30] and data are available at the site of the National Center for Complementary and Alternative medicine [NCCAM] [31]. They include conduction of electromagnetic signals, activation of opioid systems or changes in brain chemistry, sensation and involuntary body functions.

This article does not deal with the physiological effects of qi manipulation but rather with the nature of qi flow along the meridians. The hypothesis is that the transmission of qi is based on an intercellular communication system and that nitric oxide (NO) is a prime candidate to be a signaling molecule in the meridian system.

### Information and signals

Information links increase the ability to handle complexity and thus, control mechanisms in the body can be regarded as information processing. This was proposed by H. R. Maturana & F. Varela in the concept of Autopoiesis, the process by which systems organize themselves out of disorder, forming a responsive, self-maintaining network characteristic of life [32]. Autopoiesis is parallel to the concept of qi, streaming along the meridians' net, regulating our well being.

It is expected that the qi type signal molecule in the body, will be an essential entity and probably a gas so that it can spread as well as dissipate very quickly to allow following information to be transferred. Furthermore, the dual nature of NO [33,34], being either beneficial or detrimental, is parallel to the dual nature of qi [19] and fits the basic character of a signal. Signals can not always be beneficial, in that "cost" must be associated with them to ensure their "integrity", e.g., those who wish to transmit information about their richness, are likely to exhibit it by expensive jewels or costly cars, signals which are hazardous to poor people who can't afford them [35,36]. With biochemical signals, such integrity can be attained by variety of costly characters, see for example cAMP and Ca2+ [37] or Glutamate and GABA [38]. One of such characters may be the toxicity of a molecule like NO which demands very cautious and strict handling. NO differs from other neurotransmitters in that its levels are regulated solely by synthesis, rather than by storage, release or targeted degradation. NO is impossible to live without, short lived, highly diffusible and toxic [39] and is thus an excellent candidate for a cellular communication signal which carries the qi information.

## Conclusion

### What is known and what needs to be studied

The proposal presented here of NO as the carrier of qi along the meridians already has initial scientific support. Data are being accumulated indicating the tight relations between NO and acupoint manipulation. First, in rats, NO has been shown to play a role in mediating the cardiovascular responses to electroacupuncture at point 36ST [40,41], Second, acupuncture was shown to modulate the expression of NOS under diabetic conditions [42,43]. Additional experiments demonstrated that acupuncture increased the level of iNOS mRNA in macrophages [44] and while examining the distributions of NO in the skin points of rats it was shown that NO contents and nNOS expression were consistently higher in the skin acupoints/meridians associated with low electric resistance [45]. Together these data show that NO is associated with the functions of acupoint/meridian including their low electric resistance.

An initial human clinical study was performed in China and showed that the NO contents of peripheral blood increased significantly after acupuncture/moxibustion at point 36ST [46].

It is important to note that because NO is involved in multiple body functions, its presence in the peripheral blood or in an acupuncture point is a supportive but not a sufficient proof for the hypothesis.

In general a necessary approach in this case is to incorporate into the NO measures the interrelations of the meridian points based on the Chinese medicine principles [19,47]. Manipulating a distant point on the meridian and measure NO in another part of the body, or blocking NO in one part of the body and measure inhibition of a distant meridian manipulation, will demonstrate the meridian net via the NO biochemistry and the essentiality of NO in the control mechanisms of Chinese medicine.

### Final words

Traditional Chinese medicine, which was created over 2500 years ago, can play the key role in the formation of a novel integrative medicine. It can be characterized as holistic with an emphasis on the integrity of the human body and its close relationship with the environment. It is interesting to note that ancient mummies (stone aged) were discovered in Europe and in Peru, these mummies have tattoo lines partly overlapping meridians [48], so it seems that not only the Chinese have noticed this biological net.

The elucidation of the molecular basis of qi will open a vast opportunity to integrate the Chinese medical philosophy with current biological research on cellular communication, as well as with information technology in general, a theme which has become one of the main issues of our time [49,50]. Moreover, If NO is indeed a qi transmitter, the Chinese knowledge can be used as a clue to the sought after framework for NO [8] in Western medicine and further establish the link between Chinese and Western medicines. Are meridians the generic net of life? Are the major acupoints it's hubs? [51].
